# Synthesis and structural elucidation of a novel bis-spirooxindole from isatin and ethylenediamine

**DOI:** 10.3762/bjoc.22.63

**Published:** 2026-05-27

**Authors:** Irene Moreno-Gutiérrez, Josefa L López-Martínez, Sonia Berenguel-Gómez, Irene Torres-García, Duane Choquesillo-Lazarte, Manuel Muñoz-Dorado, Miriam Álvarez-Corral, Ignacio Rodríguez-García

**Affiliations:** 1 Organic Chemistry, University of Almería, CIAIMBITAL, 04120 Almería, Spain; 2 Laboratorio de Estudios Cristalográficos, IACT-CSIC. Avda. de las Palmeras 4, 18100 Armilla (Granada), Spain

**Keywords:** ethylenediamine, isatin, spirooxindole

## Abstract

The reactivity of isatin toward ethylenediamine displays an unexpected stoichiometric divergence, affording either the anticipated diiminoisatin or a previously unreported pentacyclic bis-spirooxindole. A 2:1 isatin-to-diamine ratio provides the diiminoisatin, whereas a 1:2 ratio leads to the formation of a highly symmetric bis-spirooxindole scaffold. The new spirocyclic product was fully characterized by HRMS, IR and extensive 1D/2D NMR analysis, and its structure was unequivocally established by single-crystal X-ray diffraction. In addition, the isolated diiminoisatin can be independently reduced to the same bis-spirooxindole. These results broaden the scope of isatin–diamine condensations and demonstrate their potential to generate structurally complex spirooxindole architectures under simple conditions.

## Introduction

Isatin (**1**) is a highly versatile platform for heterocycle construction, particularly through skeletal editing, ring expansion, and related transformations that enable rapid access to complex molecular architectures [1]. In parallel, the design and synthesis of spiro-heterocycles – especially spirooxindoles – has continued to expand due to their broad pharmacological relevance and the wide array of modern synthetic strategies now available for their construction, including metal-free, organocatalytic, and transition-metal-mediated approaches [2–5]. In this context, comprehensive medicinal chemistry studies consistently highlight the prominence of isatin-derived frameworks among bioactive and anticancer agents [6].

**Figure 1:**
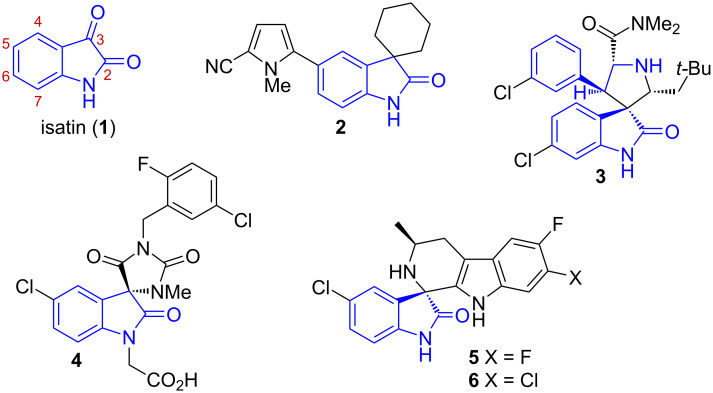
Isatin and representative bioactive spiro-fused 2-oxindoles.

The remarkable reactivity of the isatin scaffold arises from its dual functionality, combining an electrophilic C-3 carbonyl group with a γ-lactam system, which enables participation in a wide range of nucleophilic additions, condensations, and cyclizations [7]. As a result, isatin has proven to be a particularly powerful synthon for the construction of 2-oxindole derivatives, including a large variety of spiro-fused architectures. In this context, spirooxindoles have attracted considerable attention due to the conformational rigidity imparted by the spiro junction and the associated three-dimensionality, features that are often correlated with enhanced biological activity. Representative examples of bioactive spirooxindoles include the progesterone receptor modulator **2** [8], the potent MDM2–p53 inhibitor **3** [9], the CRTH2 (DP2) antagonist **4** [10] or the antimalarial agents **5** and **6** [11–12] (Figure 1). Beyond these well-known cases, several additional biologically active spirooxindoles – such as vasopressin-2 receptor antagonists, HIV-1 NNRTIs, and modulators of actin-dependent growth arrest – further exemplify the pharmacological breadth of this structural class [13–15].

**Scheme 1:**
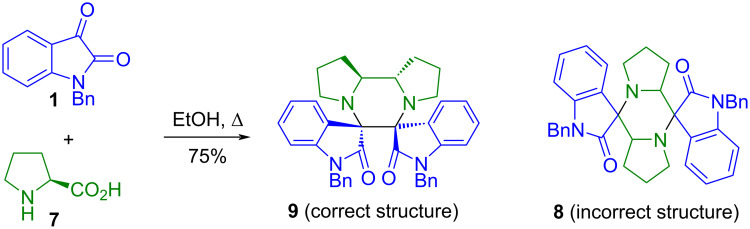
Reaction of isatin (**1**) with ʟ-proline (**7**) [16–17].

The propensity of isatin to engage in condensations with bifunctional nucleophiles has, however, also revealed a tendency toward structural complexity and, in some cases, unexpected reaction outcomes. In reactions involving amino acids or diamines, the coexistence of multiple nucleophilic and electrophilic sites can give rise to competing pathways and polycyclic architectures. A well-documented example is the condensation of isatin (**1**) with ʟ-proline (**7**), which leads to the formation of the dispirocyclic product **9** whose structure was ultimately established by X-ray diffraction after initial ambiguity based on spectroscopic data alone (Scheme 1) [16–17]. This case illustrates how condensations of isatin with bifunctional nitrogen nucleophiles may proceed beyond simple imine formation and generate highly condensed frameworks.

**Scheme 2:**
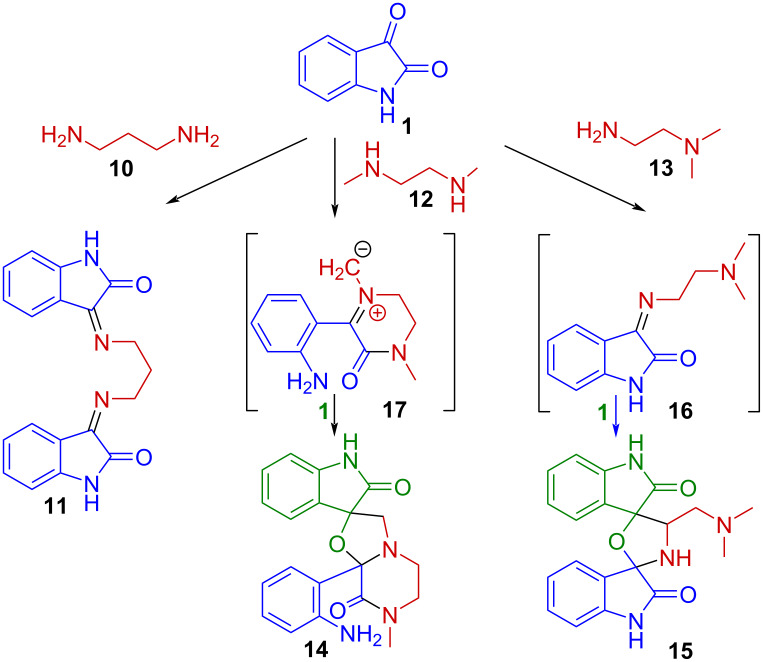
Condensations of isatin with primary [18] and *N*-methylated diamines [19].

A related behavior has been observed in reactions of isatin with diamines. Whereas condensation with propan-1,3-diamine (**10**) under controlled stoichiometry affords the corresponding diiminoisatin **11** (Scheme 2) [18], reactions involving *N*-methylated ethylenediamines like **12** or **13** lead to spirocyclic products (**14** and **15**) [19]. These outcomes were rationalized through the involvement of imine and dipolarophilic intermediates (**16** and **17**), highlighting the sensitivity of the system to substitution at nitrogen.

**Scheme 3:**
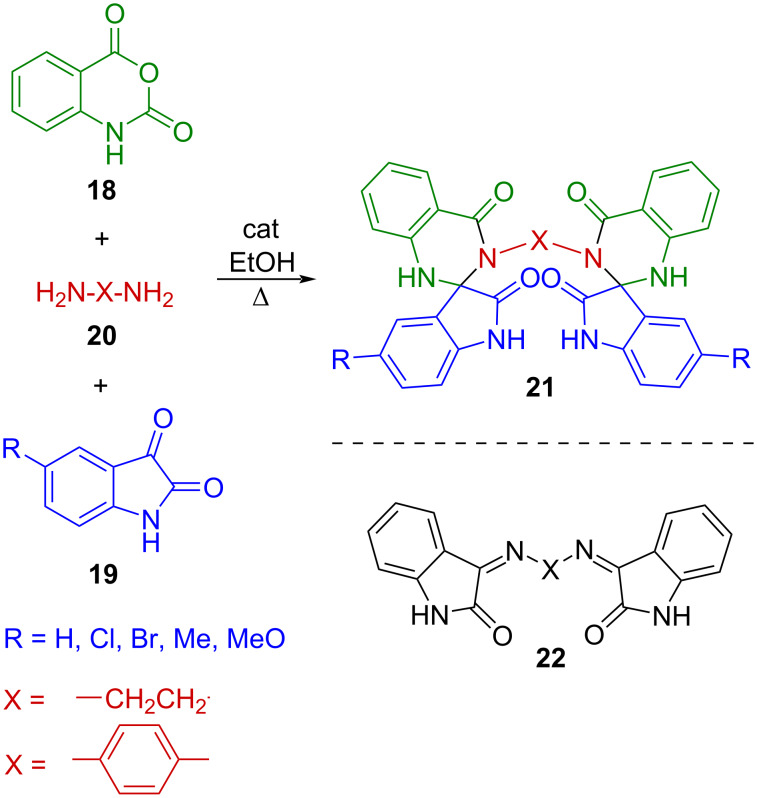
Catalyzed synthesis of the bis[spiro(quinazoline-oxindole)] derivative **21** [20].

Furthermore, multicomponent systems combining isatin derivatives with diamines have been reported to furnish bis-spirocyclic architectures like **21** without detectable formation of the corresponding diimines **22** [20] (Scheme 3).

Collectively, these precedents demonstrate that condensations of isatin with bifunctional nitrogen nucleophiles are highly sensitive to subtle variations in the nucleophile structure and reaction conditions, and may lead to markedly divergent outcomes. While simple diimines are often formed under controlled stoichiometry, alternative pathways can give rise to unexpectedly condensed spirocyclic architectures. In this context, we turned our attention to the reaction of isatin with unsubstituted ethylenediamine. Although analogy with related diamines would suggest preferential diiminoisatin formation, our results reveal a pronounced divergence in reactivity under different conditions, prompting a detailed structural investigation of the products obtained.

## Results and Discussion

**Scheme 4:**
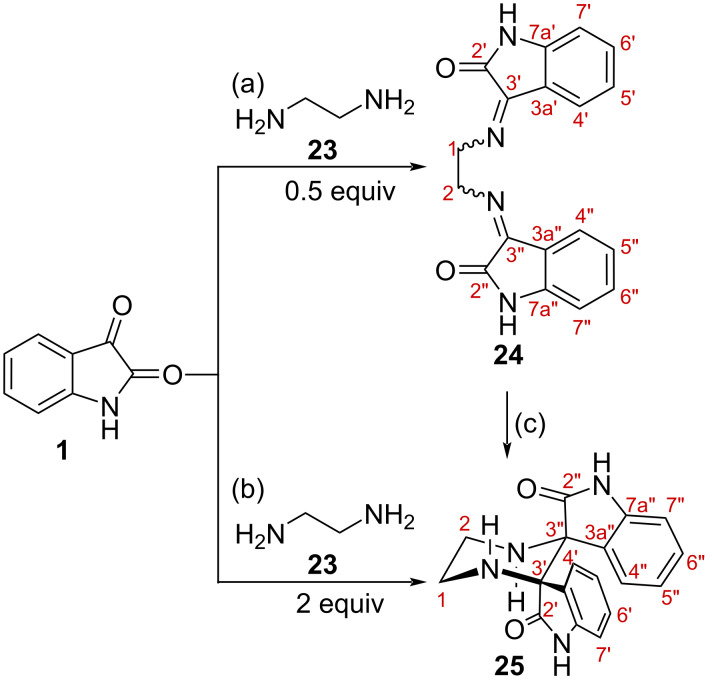
(a) Ethylenediamine (0.5 equiv), MeOH, reflux, 5 h, 66%; (b) ethylenediamine (2 equiv), EtOH, reflux, 6 h, 67%; (c) 2 equiv NaBH_4_ (58%) or NaBH_3_CN (76%), reflux, MeOH.

To investigate the divergent behavior of the reaction between isatin (**1**) and ethylenediamine (**23**), the system was examined under controlled stoichiometric conditions. Two distinct products were obtained depending on the isatin-to-diamine ratio (Scheme 4a,b). When isatin was reacted with ethylenediamine in a 2:1 molar ratio in methanol, the expected diiminoisatin **24** was obtained in 66% yield. The ¹H NMR spectrum displayed a diagnostic singlet at δ 4.45 ppm (4H) corresponding to the two =NCH₂ groups, together with the characteristic aromatic pattern of the isatin core. The IR spectrum showed a strong imine C=N band at 1610 cm^−1^, and the absence of the C-3 carbonyl stretching band confirmed complete condensation. The ^13^C NMR spectrum revealed multiple sets of closely related signals, consistent with the presence of three C=N geometrical isomers (*E*/*E, E*/*Z, Z*/*Z)*. These spectroscopic data are in agreement with those of previously described diiminoisatin derivatives [21].

When the reaction was carried out with an excess of ethylenediamine, a completely different product was obtained (Scheme 4b). Refluxing an ethanol solution of isatin and ethylenediamine in a 1:2 ratio afforded compound **25**, which gradually precipitated from the reaction mixture. Such precipitation may contribute to driving the transformation toward product formation, as performing the reaction in methanol resulted instead in a complex mixture rather than **25** as the major product. The spectroscopic features of **25** differ markedly from those of **24** and are consistent with a highly symmetric pentacyclic bis-spirooxindole framework. The HRMS spectrum displayed an [M + H]^+^ ion at *m*/*z* 321.1352, in agreement with the molecular formula C_18_H_17_N_4_O_2_.

The ^1^H NMR spectrum reflected the symmetry of **25**. The aliphatic region consisted of two doublets at δ 4.01 (2H) and 2.84 ppm (2H) with *J* = 9.3 Hz, corresponding to the axial and equatorial protons of a piperazine ring (H1, H2). The simple multiplicity is consistent with the presence of a *C*_2_ symmetry axis. The aromatic region retained the four-signal pattern of isatin (H4', H5', H6', H7'), slightly shifted by the new structural environment. In the ^13^C NMR spectrum, the amide carbonyl appeared at δ 176.5 ppm, while a key quaternary carbon at δ 61.0 ppm (C3‘) – formerly the C-3 isatin carbonyl carbon – confirmed the formation of nitrogen-bearing spiro centers. The piperazine methylenes resonated at δ 38.1 ppm, consistent with the proposed structure. The remaining signals have been assigned with the help of the two-dimensional COSY, HMBC and HSQC spectra. Altogether, these data strongly supported a bis-spirooxindole architecture.

**Figure 2:**
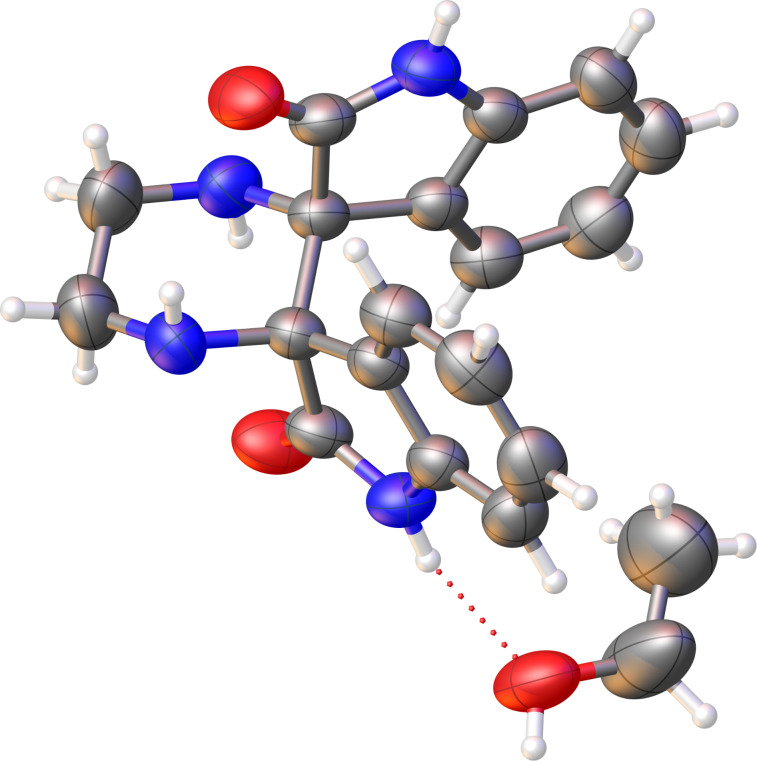
ORTEP representation of bis-spirooxindole **25** (ellipsoids at 50% probability), showing the *C*_2_-symmetric arrangement of the two oxindole units and the central piperazine ring.

Single-crystal X-ray diffraction analysis confirmed the structure of **25** unambiguously. The ORTEP representation (Figure 2) shows two oxindole units arranged orthogonally and connected by a central six-membered piperazine ring, with both isatin C-3 atoms converted into nitrogen-bearing spiro centers. The molecule displays *C*_2_ symmetry, consistent with the simplified NMR spectra, and the solid-state metrics support the proposed pentacyclic structure.

Compound **25** (C_18_H_17_N_4_O_2_ u.d. = 13) has one degree less of unsaturation than the diimine **24** (C_18_H_14_N_4_O_2_ u.d. = 14) suggesting that a reduction is required for its formation. Indeed, when **24** was treated with either sodium borohydride or sodium cyanoborohydride in methanol, compound **25** was obtained as the sole isolable product (Scheme 4c). These experiments demonstrate that **25** can arise from **24** through an initial imine reduction followed by intramolecular C–C bond formation and cyclization.

**Scheme 5:**
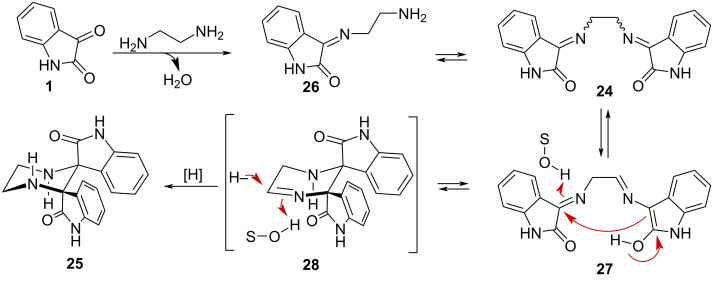
Proposed mechanism for the formation of **25**.

Scheme 5 depicts a hypothetical rationalization of the process. Reaction of isatin (**1**) with ethylenediamine (**23**) should initially form the imine **26**. Its reaction with a second molecule of isatine should lead to the diimine **24**. Under the reaction conditions both imines **26** and **24** may coexist in equilibrium. The diimine **24** could also be in equilibrium with its tautomeric form **27**. If the enol present in **27** attacks intramolecularly the other imine, it would produce a non-stabilised imine **28**, which would be electrophilic and hence susceptible to reduction either by the NaBH_4_ or by the ethylenediamine acting as a hydride donor. In addition, if the formation of **28** by addition of the enol of **27** to the imine were reversible (and the reduction step irreversible), then the more stable, dipole-opposed *C*_2_-symmetric structure for **28** should be dominant, thus explaining the observed relative stereochemistry in **25**. However, alternative pathways, cannot be excluded.

**Scheme 6:**
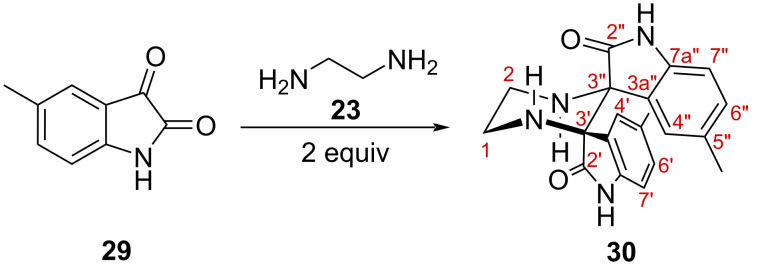
Synthesis of **30** from 5-methylisatin (**29**).

To address the generality of this transformation, a small set of substituted isatins was examined under the conditions used for the formation of **25**. Reactions with 5-bromoisatin and 5-nitroisatin led to complex mixtures from which no defined bis-spirooxindole product could be isolated. In contrast, 5-methylisatin (**29**) afforded the corresponding methyl-substituted bis-spirooxindole **30**, indicating that the transformation is not restricted to unsubstituted isatin, although it appears to be sensitive to the electronic nature of the substrate. This preliminary exploration therefore suggests that electron-neutral or weakly electron-donating substrates may be more suitable for this process, whereas strongly electron-withdrawing or halogenated derivatives require further optimization (Scheme 6).

Taken together, these results show that the reaction of isatin with ethylenediamine can be directed toward different structural outcomes depending on stoichiometry. Simple control of stoichiometry governs whether the system evolves through a conventional diimine pathway or diverges toward the assembly of a highly condensed bis-spirooxindole framework.

## Conclusion

The condensation of isatin with ethylenediamine is strongly governed by stoichiometry, allowing selective access either to a diiminoisatin **24** or to the unexpected pentacyclic bis-spirooxindole **25** (Scheme 4) which was unambiguously identified by comprehensive spectroscopic analysis and single-crystal X-ray diffraction. The same bis-spirooxindole **25** can be obtained by reduction of the diiminoisatin **24**. The reaction with methyl-substituted isatin **29** follows the same pattern, affording **30**. Overall, we have shown that simple isatin–diamine systems can deliver structurally complex spirocyclic architectures under mild and operationally straightforward conditions, although the substrate scope appears to be limited.

## Experimental

**General remarks:** NMR spectra were recorded on a Bruker Nanobay Avance III HD 600 MHz spectrometer. Proton-decoupled. When required, COSY, HMQC and HMBC experiments were used for signal assignment. Chemical shifts (δ) are expressed in ppm and coupling constants (*J*) in hertz (Hz). Chemical shifts are reported using CD_3_OD or DMSO-*d*_6_ as internal reference. IR spectra were recorded with a Bruker Alpha spectrometer using a single reflection ATR-platinum module. Mass spectra were recorded in a Waters Xevo LC-QTof-MS with electrospray ionization. The AQ:AcN mixture (50:50, 0.1% formic acid) was used as eluent. X-ray diffraction data were collected on a Bruker AXS SMART APEX diffractometer. Structure solution and refinement were carried out using Olex2 [22]. The structure was solved by intrinsic phasing with SHELXT [23] and refined by full-matrix least-squares methods on F^2^ using SHELXL [24]. Commercially available chemicals were obtained from Scharlau, TCI and Acros and used as received.

**Synthesis of 25:** Isatin (**1**, 2.98 g, 19.85 mmol, 1 equiv) was dissolved in ethanol (60 mL) and a solution of ethylenediamine (2.25 mL, 39.70 mmol, 2.0 equiv) in ethanol (60 mL) was added dropwise. The mixture was heated at reflux for 6 h and then allowed to crystallize at 4 °C for 72 h. The solid was collected by filtration and washed with cold ethanol to give **25** as yellow crystals (1.06 g, 6.62 mmol, 67%). Mp = 169.5–170.4 °C; HRMS *m*/*z*: [M + H]^+^ calcd. for [C_18_H_17_N_4_O_2_ + H]^+^, 321.1352; found, 321.1358; IR (ATR) *ν* (cm^−1^): 3276, 1710, 1619, 1472, 744; ^1^H NMR (600 MHz, CD_3_OD) δ (ppm) 7.32 (ddd, *J* = 7.7, 1.1, 0.6 Hz, 2H, H4’, H4’’), 7.08 (td, *J* = 7.7, 1.2 Hz, 2H, H6’, H6’’), 6.90 (td, *J* = 7.7, 1.0 Hz, 2H, H5’, H5’’), 6.79–6.77 (m, 2H, NH), 6.62 (ddd, *J* = 7.7, 0.9, 0.6 Hz, 2H, H7’, H7’’), 4.01 (d, *J* = 9.3 Hz, 2H, H1ax, H2ax), 2.84 (d, *J* = 9.3 Hz, 2H, H1eq, H2eq); ^13^CNMR (150.92 MHz, CD_3_OD) 176.5 (C, C2’, C2’’), 141.7 (C, C7’a, C7’’a), 129.2 (CH, C6’, C6’’), 127.7 (C, C3a’, C3’’a), 123.8 (CH, C4’, C4’’), 121.9 (CH, C5’, C5’’), 109.3 (CH, C7’, C7’’), 61.0 (C, C3’, C3’’), 38.1 (CH_2_, C1, C2).

**X-ray analysis of 25** [22–24]. Recrystallization of **25** in EtOH afforded a yellow crystal. Crystal data for C_22_H_26_N_4_O_4_ (*M* = 410.47 g/mol): monoclinic, space group *C*2/*c* (no. 15), *a* = 16.0100(5) Å, *b* = 8.5258(4) Å, *c* = 16.6847(5) Å, *β* = 105.815(2)°, *V* = 2191.22(14) Å^3^, *Z* = 4, *T* = 298(2) K, μ(Cu Kα) = 0.712 mm^−1^, *Dcalc* = 1.244 g/cm^3^, 11324 reflections measured (11.488° ≤ 2Θ ≤ 136.964°), 2001 unique (*R*_int_ = 0.0404, *R*_sigma_ = 0.0308) which were used in all calculations. The final *R*_1_ was 0.0595 (I > 2σ(I)) and *wR*_2_ was 0.1864 (all data). CCDC 2388389 contains the supplementary crystallographic data for this paper. These data can be obtained free of charge at https://doi.org/10.5517/ccdc.csd.cc2l59tx from the CCDC, 12 Union Road, Cambridge CB2 1EZ, UK; Fax: +44-1223-336033; E-mail: deposit@ccdc.cam.ac.uk.

**Synthesis of 24.** Ethylenediamine (0.33 mL, 5 mmol, 1 equiv) was added dropwise to a solution of isatin (**1**, 1.47 g, 10 mmol, 2 equiv) in MeOH (50 mL). The mixture was heated at reflux for 5 h, cooled to 0 °C, and acidified with 1 M HCl (to pH ≈ 1), leading to precipitation of the product. The orange precipitate was collected by filtration, washed with cold MeOH, and dried under vacuum to give **24** (1.05 g, 3.3 mmol, 66%). HRMS *m/z*: [M + H]^+^ calcd. for [C_18_H_14_N_4_O_2_ + H]^+^, 319.1190; found, 319.1188; IR (ATR) *ν* (cm^−1^): 3270, 2890, 1740, 1707, 1652, 1610, 1464, 1342, 1205, 1021, 739; ^1^H NMR (600 MHz, DMSO-*d*_6_) δ (ppm) 10.83 (bs, 2H, NH), 7.84 (bd, *J* = 7.7 Hz, 2H, H4’, H4’’), 7.45 (td, *J* = 7.8, 1.1 Hz, 2H, H6’, H6’’), 7.08 (td, *J* = 7.7, 0.9 Hz, 2H, H5’,H5’’), 6.94 (bd, *J* = 7.8 Hz, 2H, H7’,H7’’), 4.45 (s, 4H, =NCH_2_); ^13^C NMR (125 MHz, DMSO-*d*_6_) δ 164.08 (C), 164.05 (C), 160.18(C), 155.86 (C), 155.74 (C), 154.89 (C), 146.41 (C), 146.36 (C), 145.07 (C), 134.05 (CH), 133.95 (CH), 133.49 (CH), 127.84 (CH), 127.79 (CH), 122.69 (CH), 122.64 (CH), 122.63 (CH), 122.20 (CH), 117.33 (C), 117.31 (C), 111.57 (CH), 111.51 (CH), 111.05 (CH), 55.61 (CH_2_), 55.47 (CH_2_), 53.07 (CH_2_).

**Reduction of 24 with NaBH_4_.** NaBH₄ (95 mg, 2.52 mmol, 2 equiv) was added in portions to a stirred solution of **24** (0.40 g, 1.26 mmol, 1 equiv) in MeOH (15 mL) at room temperature. The mixture was stirred for 2 h, poured onto ice, and the resulting yellow precipitate of **25** was collected by filtration (295 mg, 0.92 mmol, 58%).

**Reduction of 24 with NaBH_3_CN.** NaBH_3_CN (0.16 g, 2.52 mmol, 2 equiv) was slowly added to a solution of **24** (0.4 g, 1.26 mmol, 1 equiv) in MeOH (15 mL). The reaction mixture was stirred at reflux for 1 h 30 min, poured over ice and vacuum filtered. **25** was obtained as a yellow precipitate (305 mg, 0.95 mmol, 76%).

**Synthesis of 30.** 5-Methylisatin (**29**, 1.50 g, 9.8 mmol, 1 equiv) was dissolved in ethanol (30 mL) and a solution of ethylenediamine (1.3 mL, 19.6 mmol, 2.0 equiv) in ethanol (30 mL) was added dropwise. The mixture was heated at reflux for 6 h. The solvent was removed under reduced pressure and the mixture was purified by column chromatography (DCM/Me_2_CO gradient from 8:2 to 6:4) to give **30** as yellow oil (1.04 g, 2.99 mmol, 61%). HRMS *m/z*: [M + H]^+^ calcd. for [C_20_H_21_N_4_O_2_ + H]^+^, 349.1665; found, 349.1674; ^1^H NMR (300 MHz, MeOD) δ 7.19–7.17 (m, 2H, H4’, H4’’), 6.92 (ddd, *J* = 7.9, 1.7, 0.8 Hz, 2H, H6’, H6’’), 6.70 (bs, 1H, NH), 6.68 (bs, 1H, NH),6.54 (d, *J* = 7.9 Hz, 2H, H7’, H7’’), 4.01 (d, *J* = 9.2 Hz, 2H, H1ax, H2ax), 2.84 (d, *J* = 9.2 Hz, 2H, H1eq, H2eq), 2.24 (s, 6H); ^13^C NMR (75 MHz, MeOD) δ 176.5 (C, C2’, C2’’), 139.2 (C, C7’a, C7’’a), 131.6 (C, C5a’, C5’’a), 129.5 (CH, C6’, C6’’), 127.9 (C, C3a’, C3’’a), 124.5 (CH, C4’, C4’’), 109.2 (CH, C7’, C7’’), 61.0 (C, C3’, C3’’), 38.2 (CH_2_, C1, C2), 19.9 (CH_3_).

## Supporting Information

File 1 Copies of IR, NMR and MS spectra.

## Data Availability

All data that supports the findings of this study is available in the published article and/or the supporting information of this article.
