# Supplementary material for: Synthesis and structural elucidation of a novel bis-spirooxindole from isatin and ethylenediamine
Source: Beilstein J Org Chem. 2026 May 27;22:813–20. doi: 10.3762/bjoc.22.63 (PMC13224053; doi:10.3762/bjoc.22.63)
Supplement: File 1 — Copies of IR, NMR and MS spectra. [file Beilstein_J_Org_Chem-22-813-s001.pdf]

BEILSTEIN JOURNAL OF ORGANIC CHEMISTRY

Supporting Information

for

Synthesis and structural elucidation of a novel bis-spirooxindole from isatin and ethylenediamine

Irene Moreno-Gutiérrez, Josefa L. López-Martínez, Sonia Berenguel-Gómez, Irene Torres-García, Duane Choquesillo-Lazarte, Manuel Muñoz-Dorado, Miriam Álvarez-Corral and Ignacio Rodríguez-García

Beilstein J. Org. Chem. **2026,** *22,* 813–820. doi:10.3762/bjoc.22.63

Copies of IR, NMR and MS spectra

License and Terms: This is a supporting information file under the terms of the Creative Commons Attribution License (https://creativecommons.org/licenses/by/4.0). Please note that the reuse, redistribution and reproduction in particular requires that the author(s) and source are credited and that individual graphics may be subject to special legal provisions.
The license is subject to the *Beilstein Journal of Organic Chemistry* terms and conditions: (https://www.beilstein-journals.org/bjoc/terms)

Index

Spectroscopic characterization of 25 .... **S2**
Figure S1. ^{1}H NMR (600 MHz, CD_3OD) of **25** S2
Figure S2. ^{13}C NMR and DEPT (150 MHz, CD_3OD) of **25** S3
Figure S3. 2D NMR (COSY) (600 MHz, CD_3OD) of **25** S4
Figure S4. 2D NMR (HMQC) (600 MHz, CD_3OD) of **25** S5
Figure S5. 2D NMR (HMBC) (600 MHz, CD_3OD) of **25** S6
Figure S6. HRMS Q-TOF of **25** S7
Figure S7. IR (ATR) of **25** S8
Spectroscopic characterization of 24 .... **S9**
Figure S8. ^{1}H NMR (600 MHz, DMSO-d_6) of **24** S9
Figure S9. ^{13}C NMR and DEPT (151 MHz, DMSO-d_6) of **24** S10
Figure S10. IR (ATR) of **24** S11
Spectroscopic characterization of 30 .... **S12**
Figure S11. ^{1}H NMR (300 MHz, CD_3OD) of **30** S12
Figure S12. ^{13}C NMR and DEPT (75 MHz, CD_3OD) of **30** S13
Figure S13. 2D NMR (COSY) (300 MHz, CD_3OD) of **30** S14
Figure S14. 2D NMR (HMQC) (300 MHz, CD_3OD) of **30** S15
Figure S15. 2D NMR (HMBC) (600 MHz, CD_3OD) of **30** S16

Spectroscopic characterization of 25

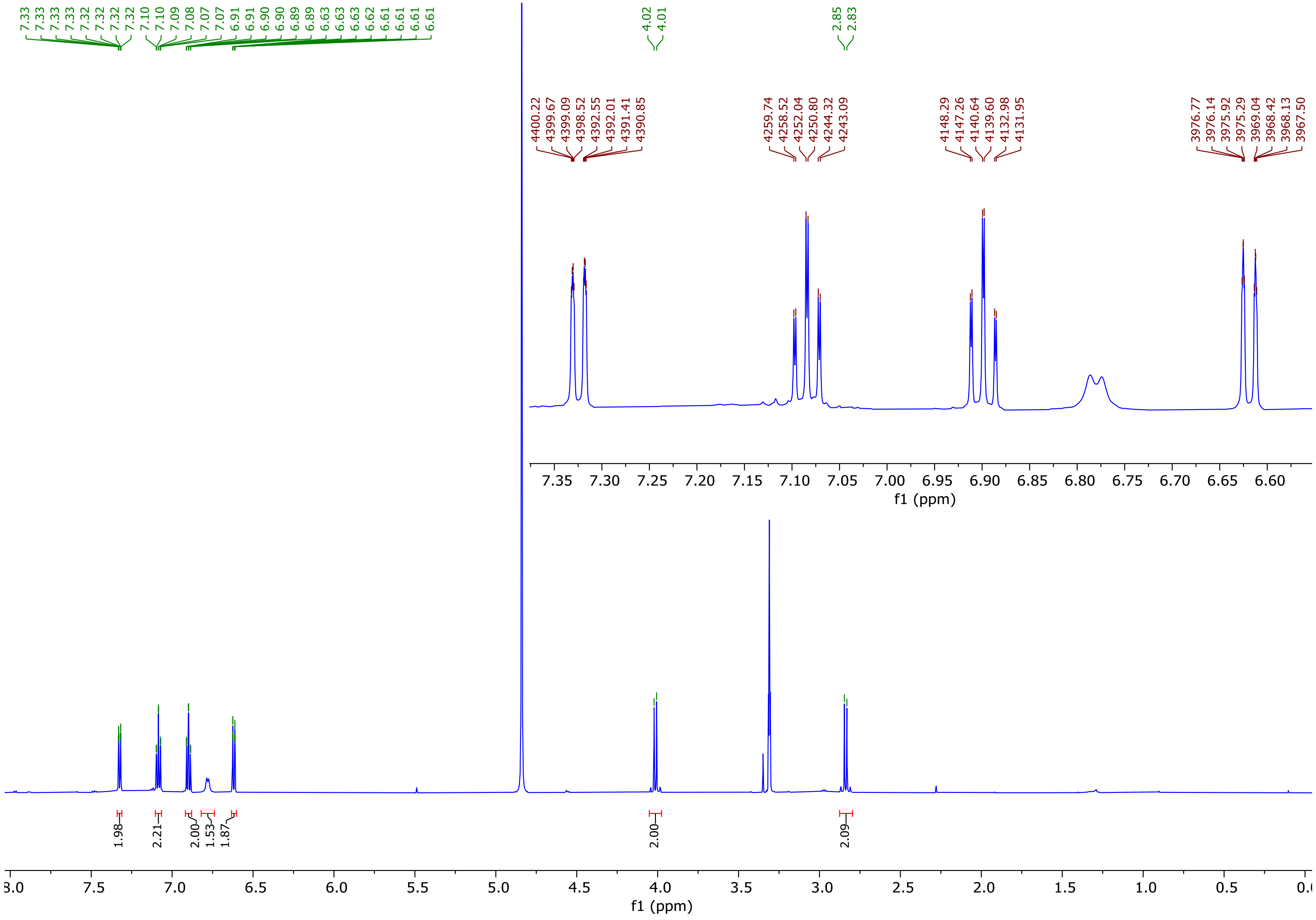

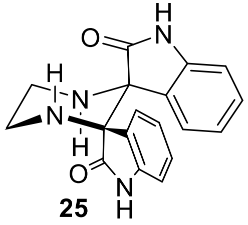

25

Figure S1. ^{1}H NMR (600 MHz, CD_3OD) of **25**.

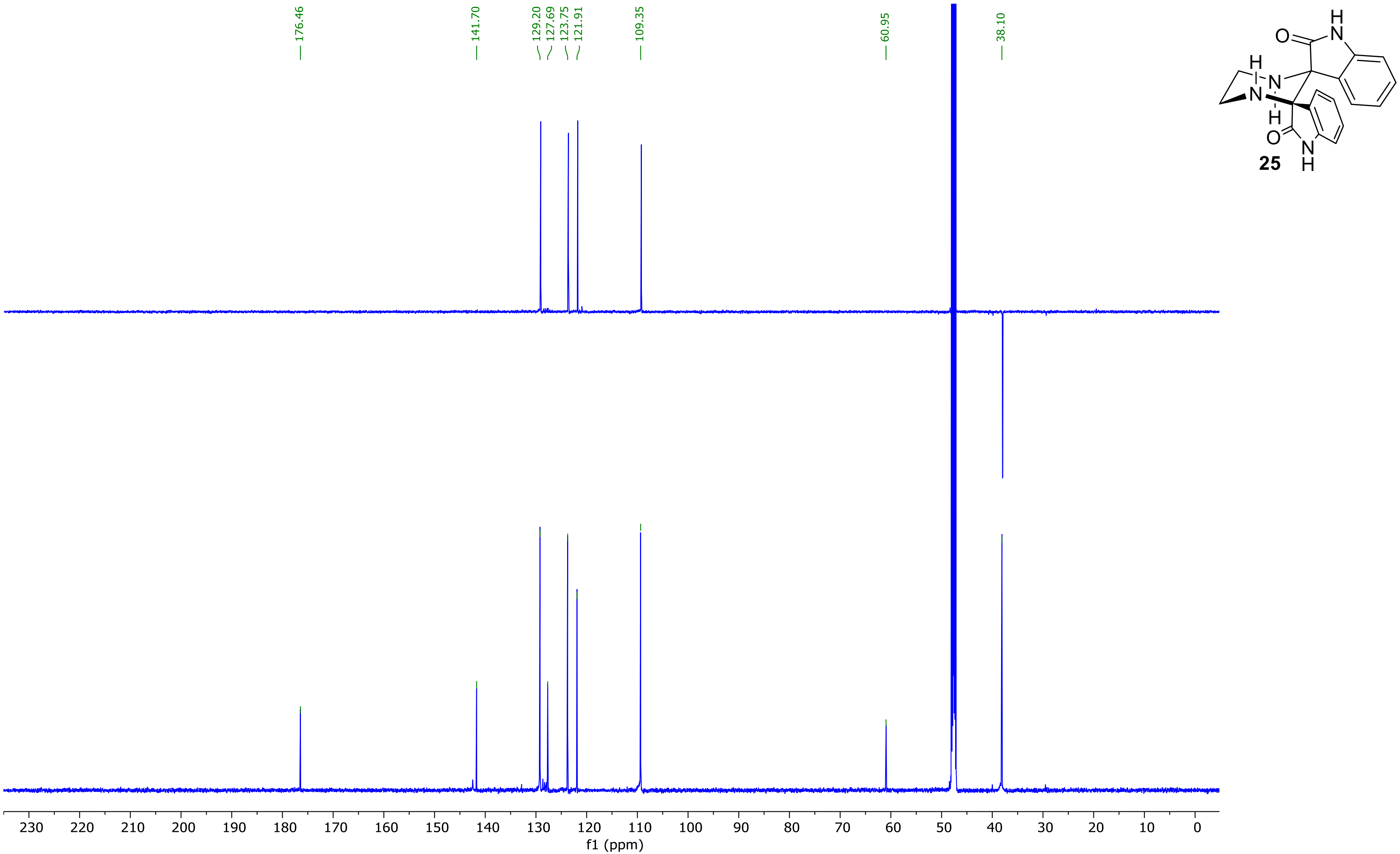

Figure S2. ^{13}C NMR and DEPT (150 MHz, CD_3OD) of **25**.

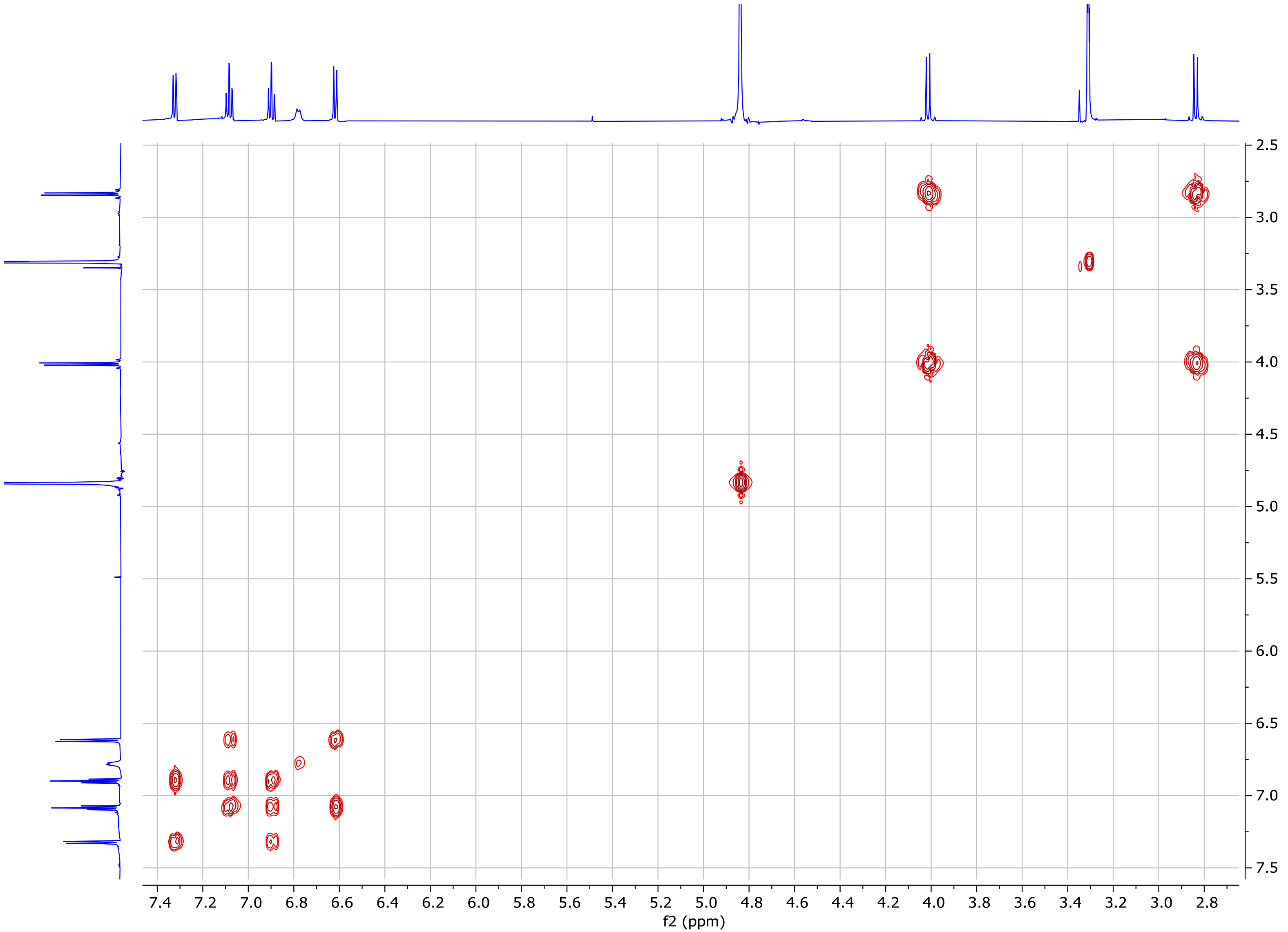

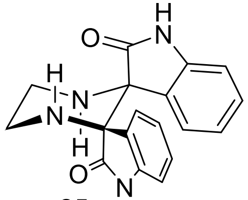

25

Figure S3. 2D NMR (COSY) (600 MHz, CD_3OD) of **25**.

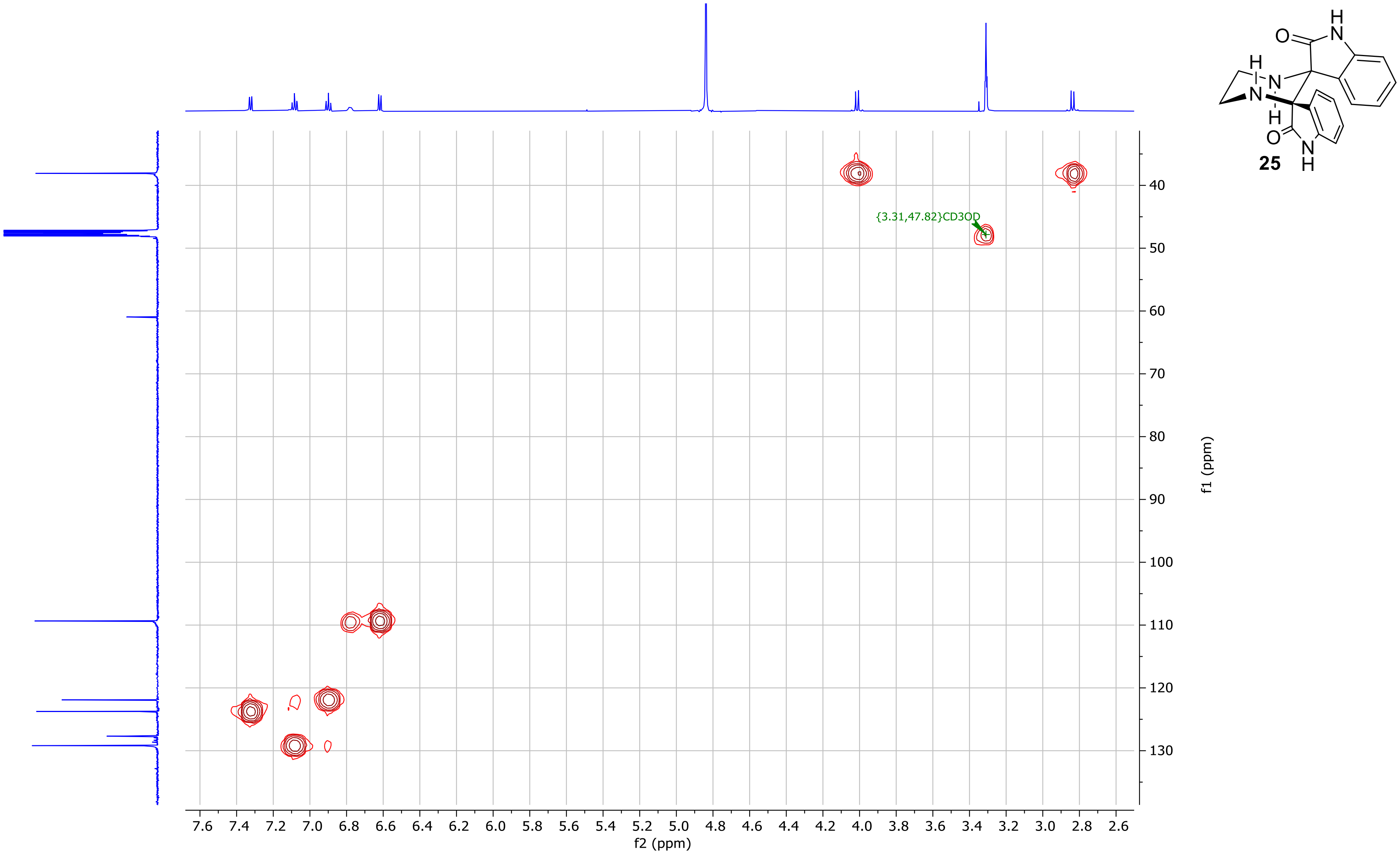

Figure S4. 2D NMR (HMQC) (600 MHz, CD_3OD) of **25**.

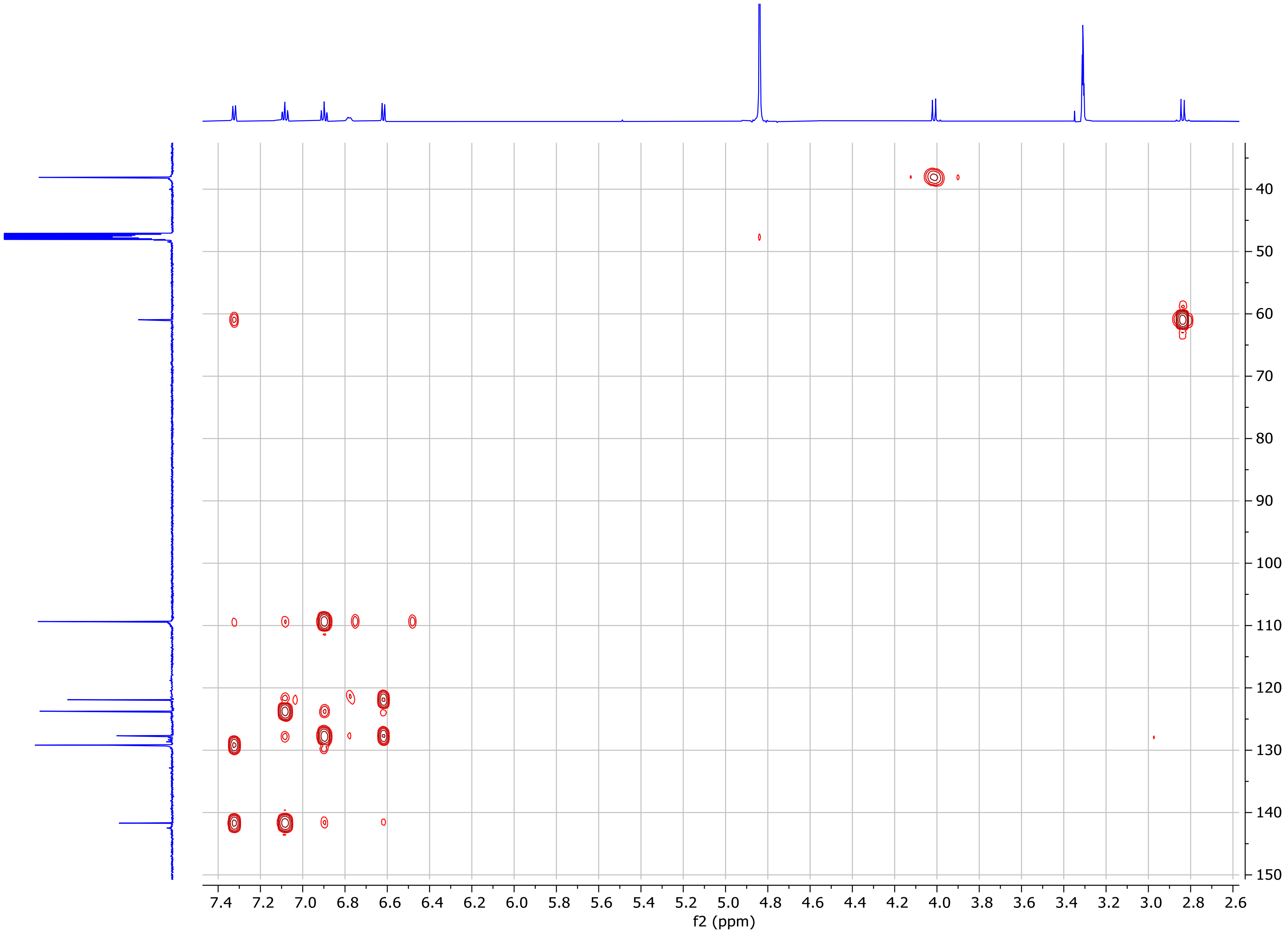

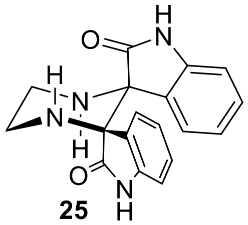

25

Figure S5. 2D NMR (HMBC) (600 MHz, CD_3OD) of **25**.

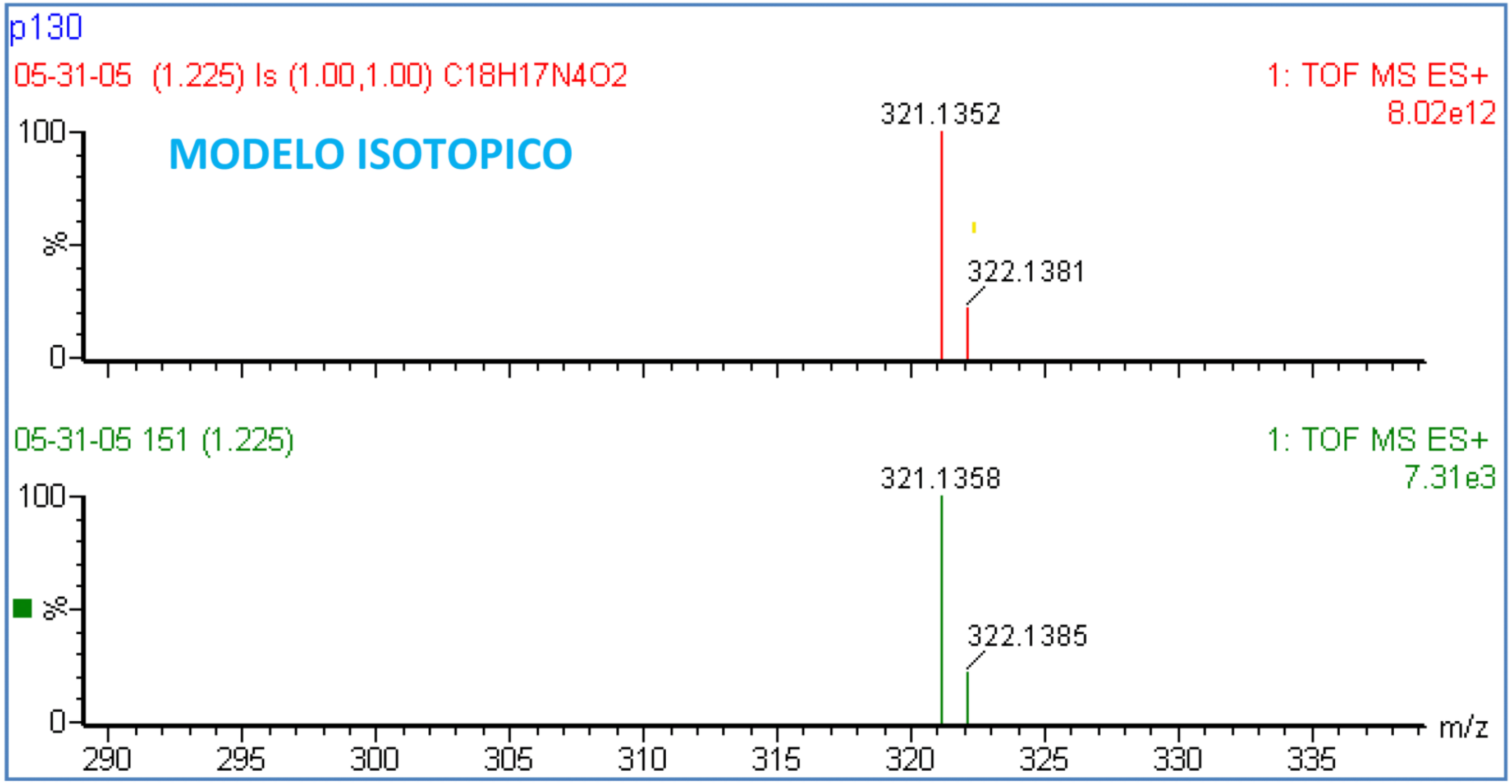

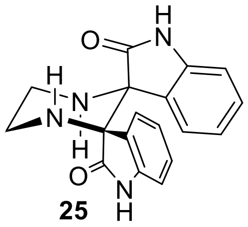

25

Figure S6. HRMS Q-TOF of **25**.

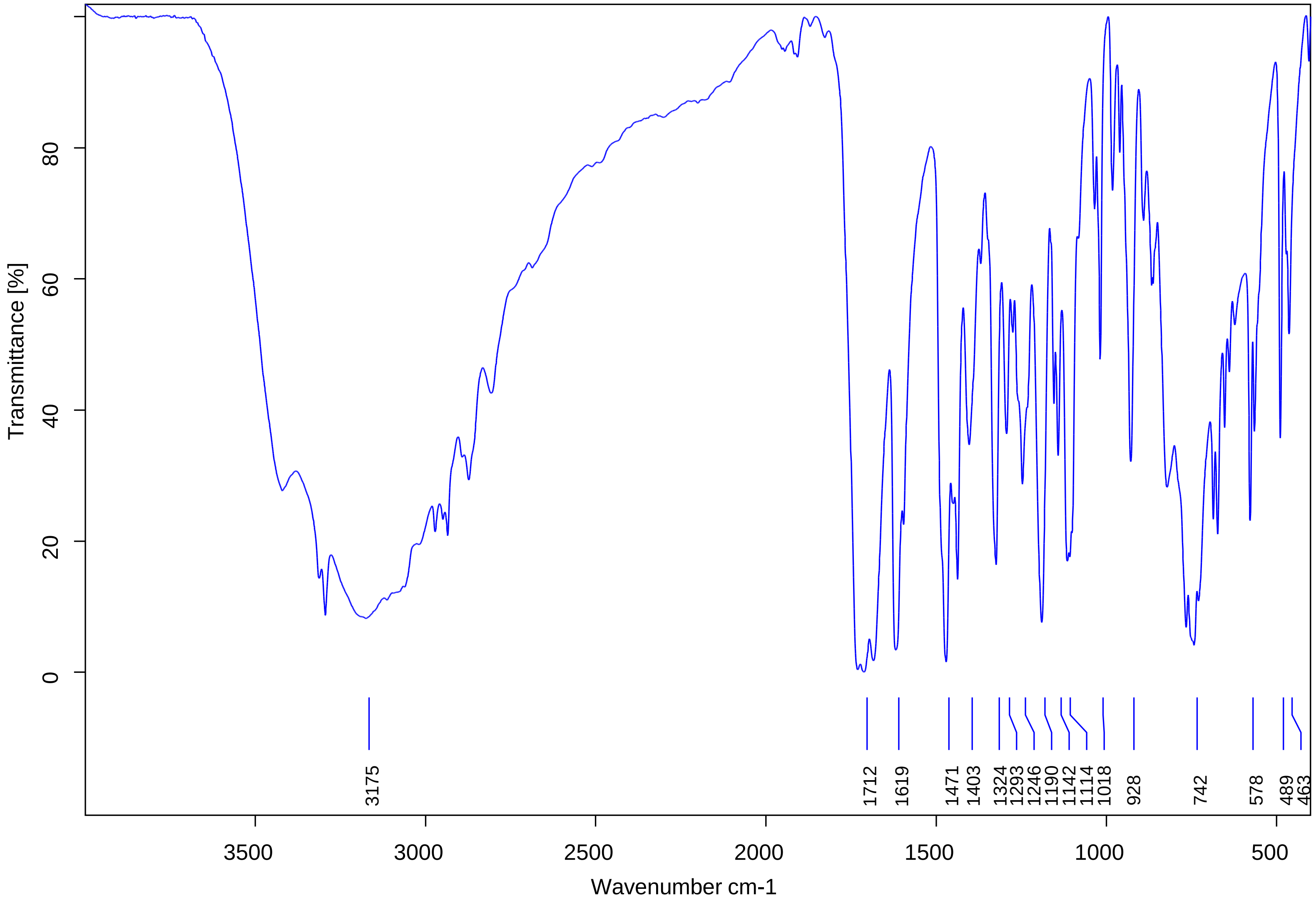

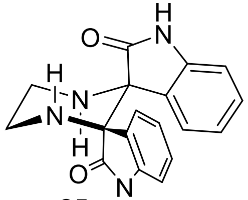

25

Figure S7. IR (ATR) of **25**.

Spectroscopic characterization of 24

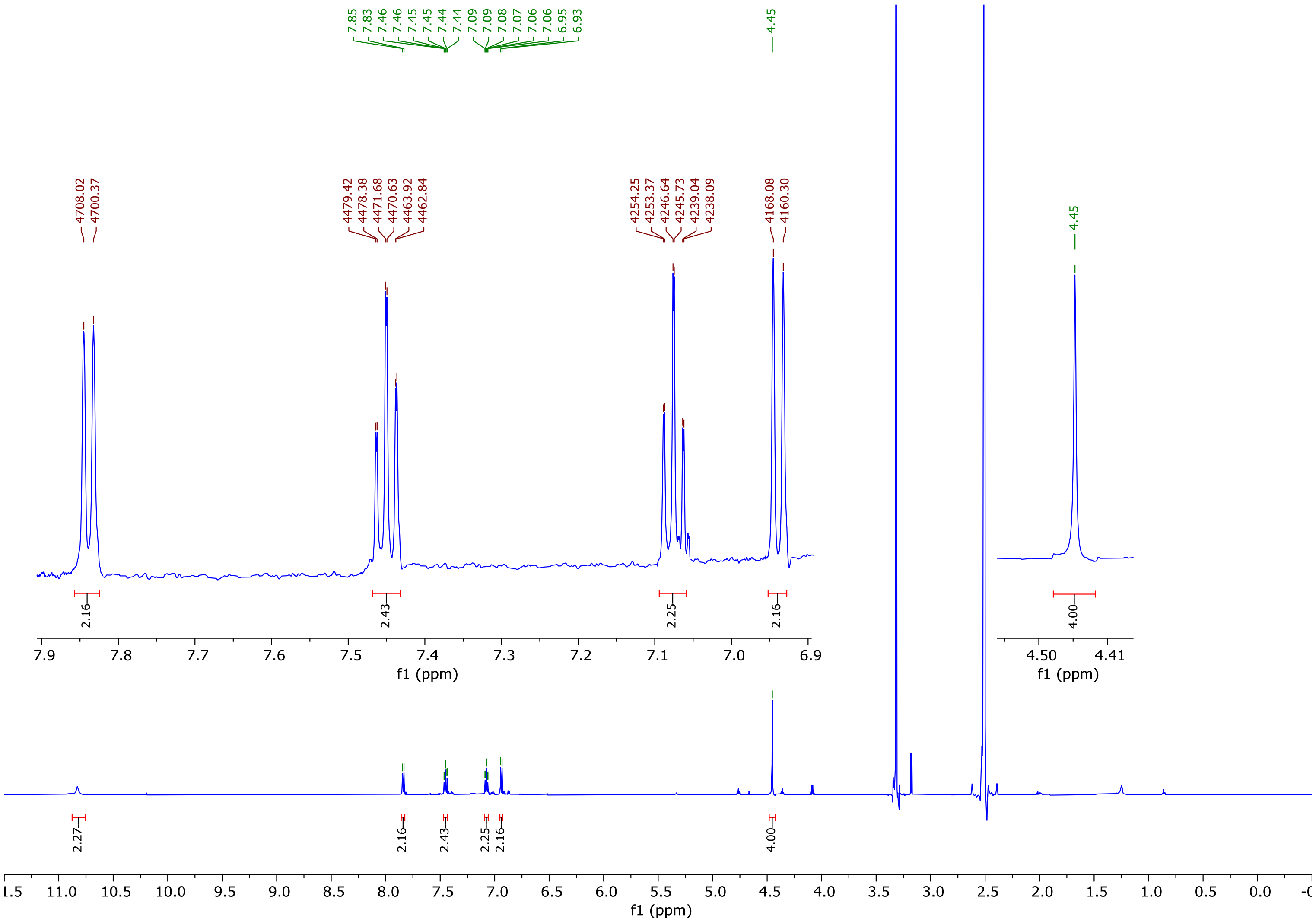

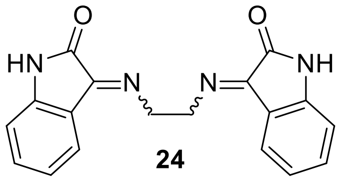

24

Figure S8. ^{1}H NMR (600 MHz, DMSO-d$_6$) of **24**.

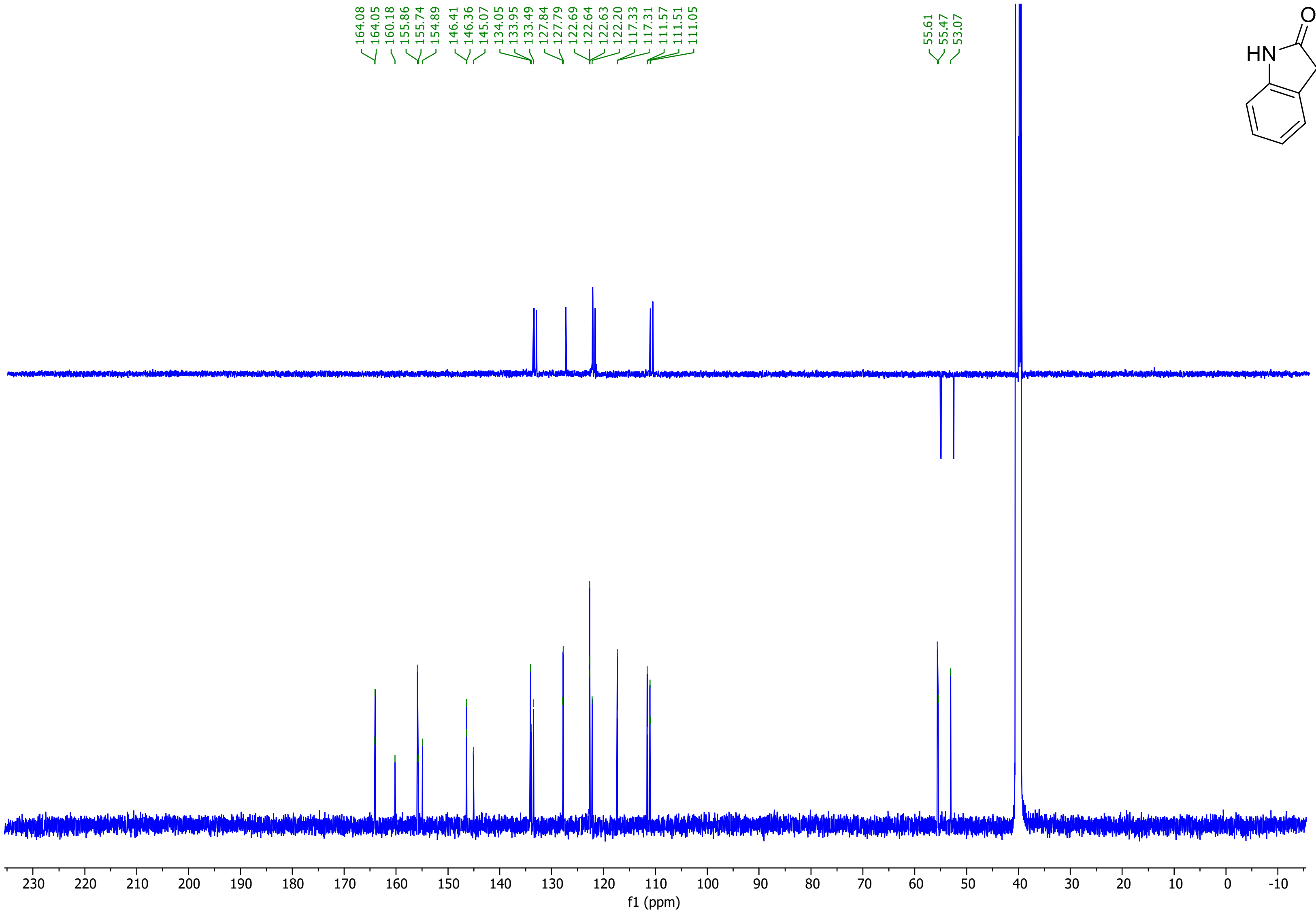

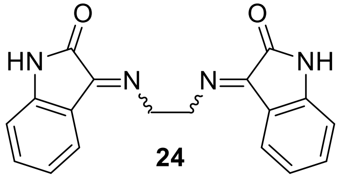

24

Figure S9. ^{13}C NMR and DEPT (151 MHz, DMSO-d_6) of **24**.

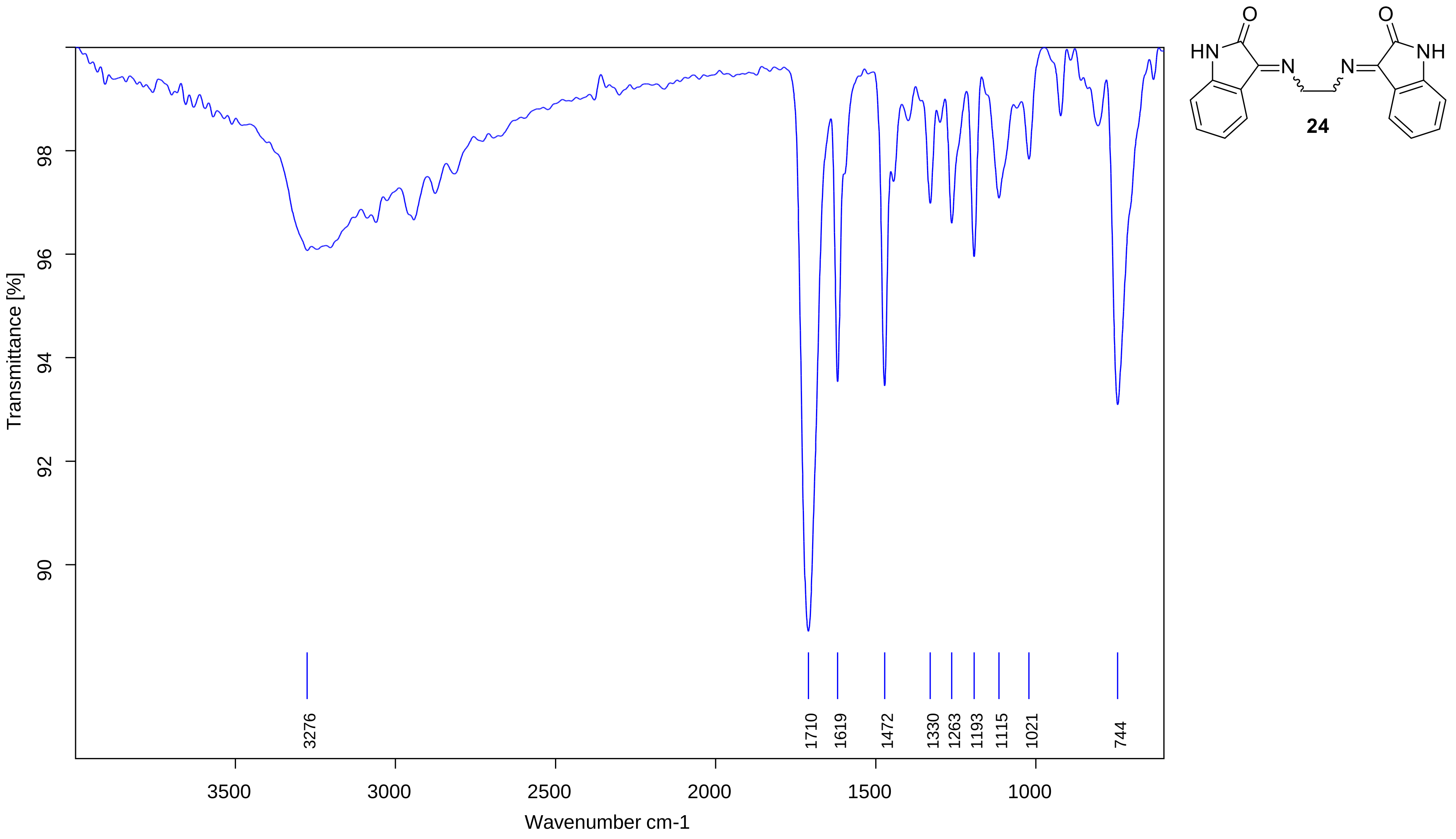

Figure S10. IR (ATR) of **24**.

Spectroscopic characterization of 30

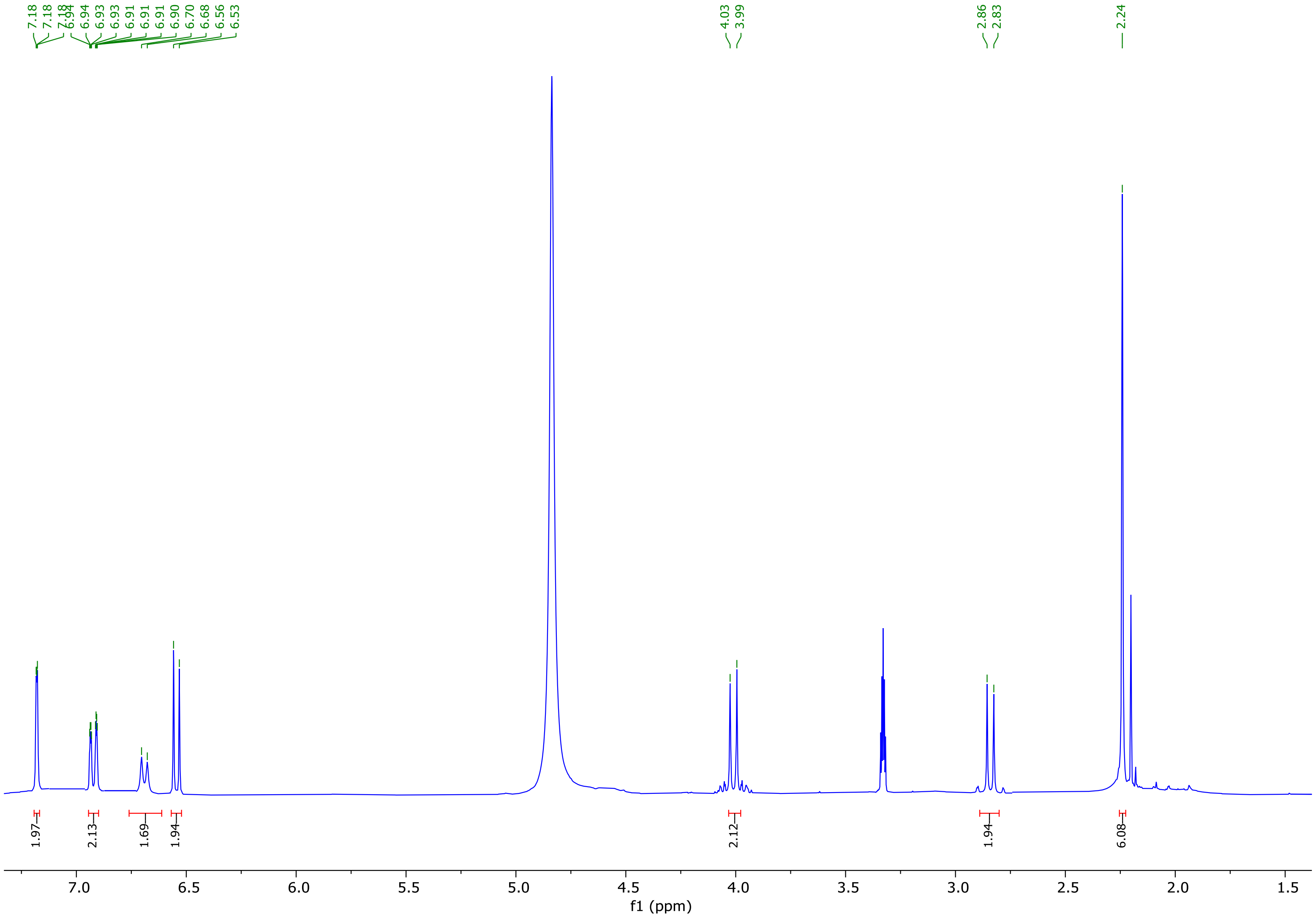

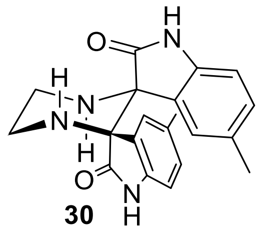

30

Figure S11. ^{1}H NMR (300 MHz, CD_3OD) of **30**.

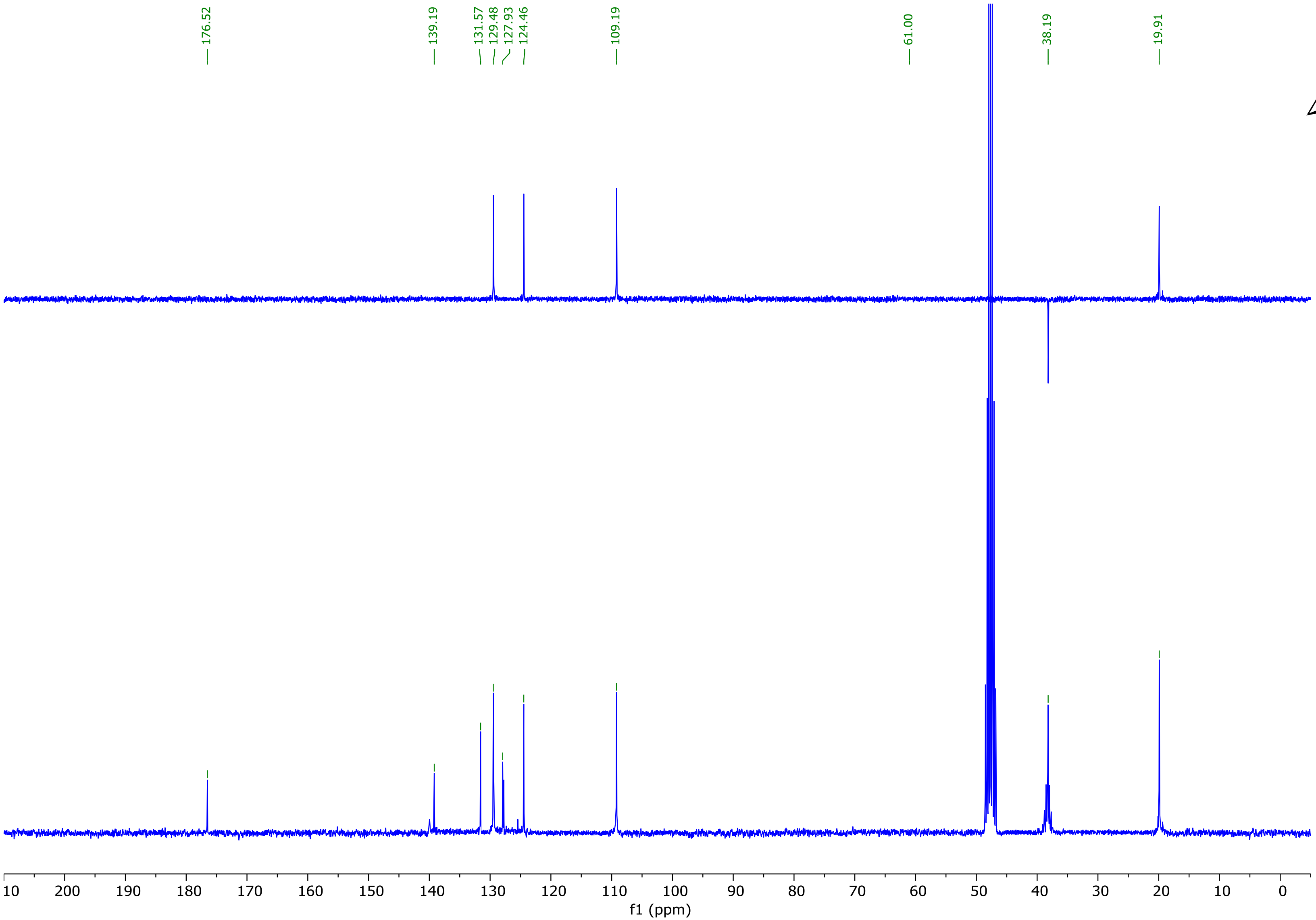

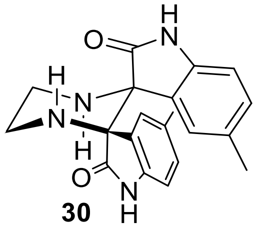

30

Figure S12. ^{13}C NMR and DEPT (75 MHz, CD_3OD) of **30**.

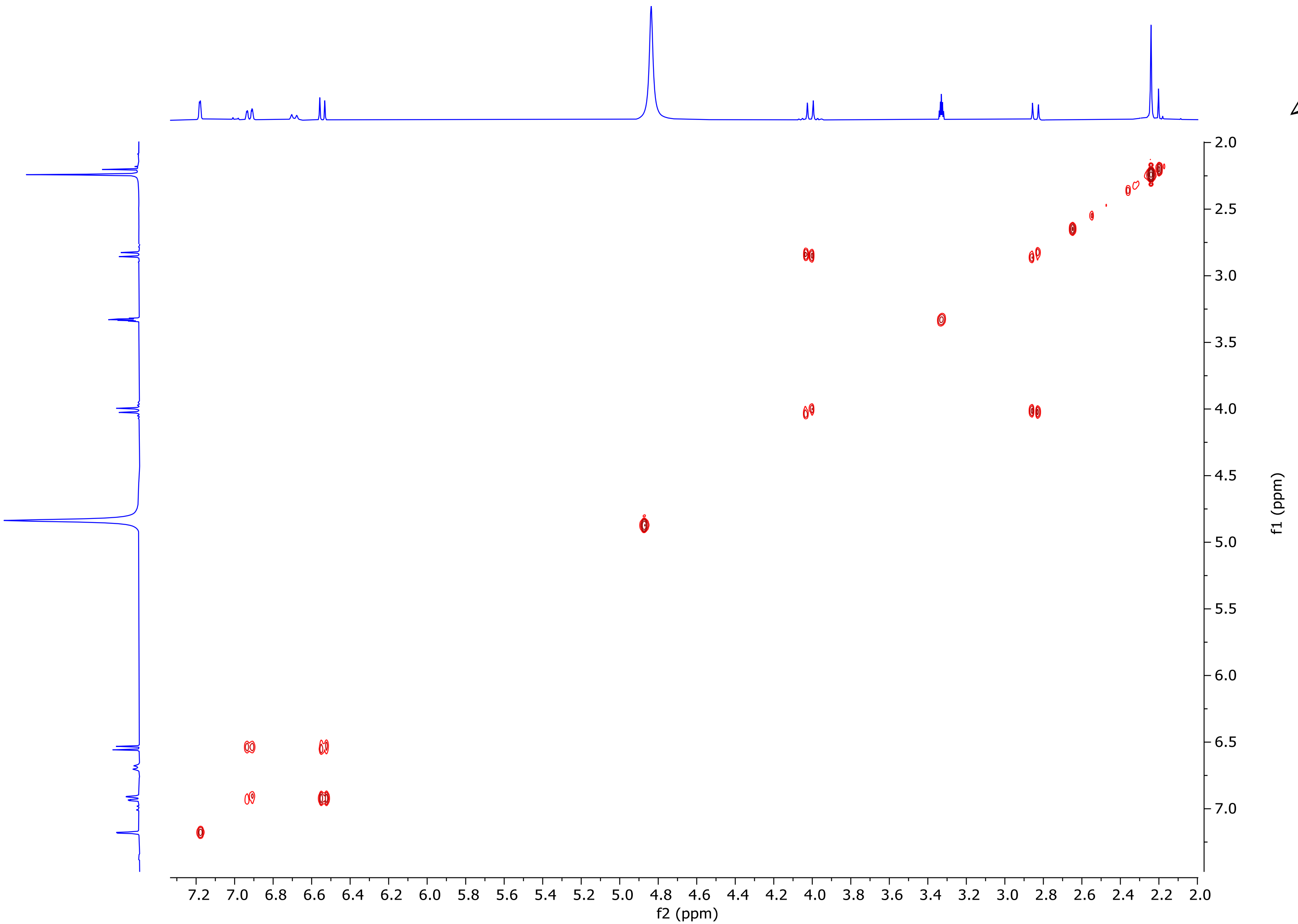

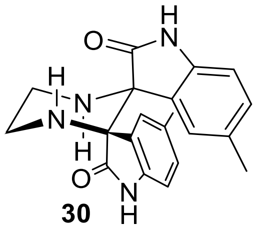

30

Figure S13. 2D NMR (COSY) (300 MHz, CD_3OD) of **30**.

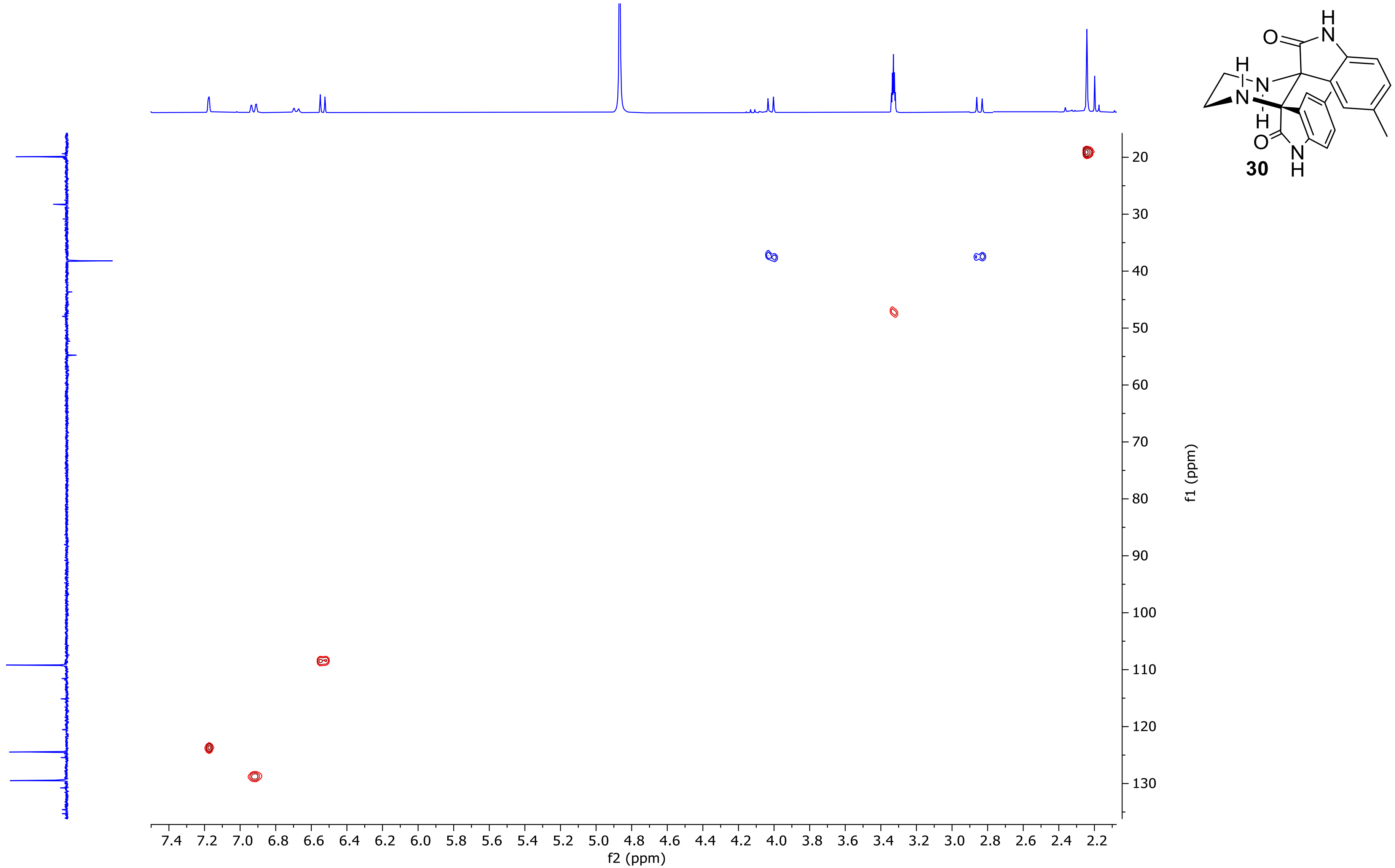

30

Figure S14. 2D NMR (HMQC) (300 MHz, CD_3OD) of **30**.

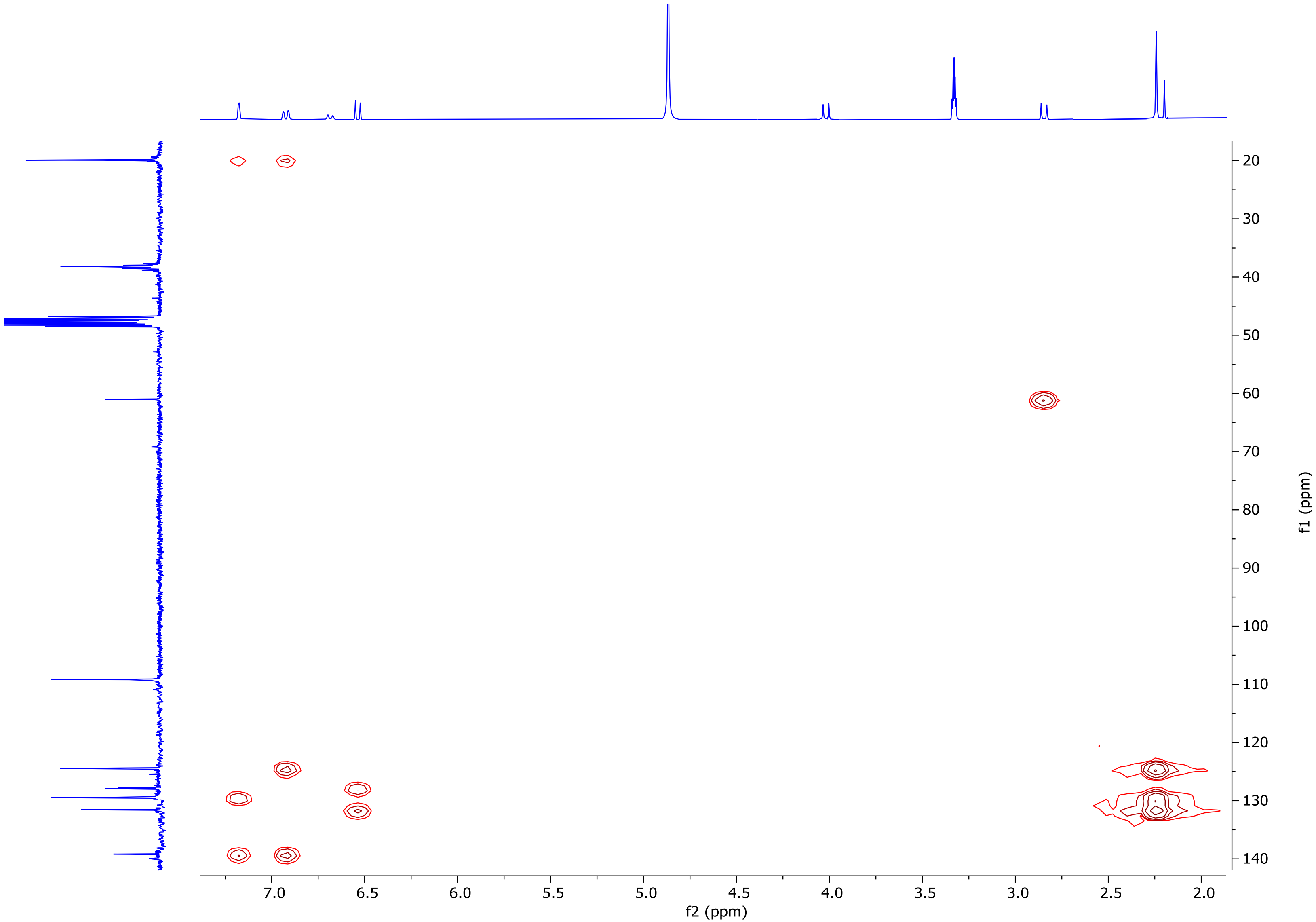

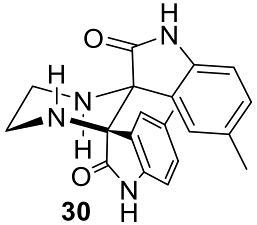

30

Figure S15. 2D NMR (HMBC) (600 MHz, CD_3OD) of **30**.
